# Gradual Morphological Tuning in Polymer Microspheres via Pickering Emulsion Synthesis: Architecture-Controlled Dye Adsorption and Encapsulation

**DOI:** 10.3390/ijms27062591

**Published:** 2026-03-12

**Authors:** Mirela Honciuc, Oana-Iuliana Negru, Andrei Honciuc

**Affiliations:** “Petru Poni” Institute of Macromolecular Chemistry, 41A Grigore Ghica Voda Alley, 700487 Iasi, Romania; negru.oana@icmpp.ro (O.-I.N.); honciuc.andrei@icmpp.ro (A.H.)

**Keywords:** Pickering emulsion polymerization, porous polymer microspheres, hierarchical porosity, adsorption kinetics, methylene blue adsorption, diffusion–kinetic modeling, dye removal

## Abstract

Polymeric microspheres synthesized via Pickering emulsion polymerization offer structural tunability, making them attractive platforms for dye adsorption. This study investigates the adsorption behavior of methylene blue onto two classes of polymeric microspheres—poly(methacrylic acid) crosslinked with ethylene glycol dimethacrylate (PM), containing both micro- and nanopores, and poly(methacrylic acid) crosslinked with divinylbenzene (PD), containing only nanopores. The adsorption kinetics were modeled using a dual-process approach that distinguishes between diffusion-controlled transport and surface-controlled kinetic adsorption. We quantified the relative contributions of these mechanisms and correlated them with particle architecture. In the PM particles, diffusion plays a significant role in smaller particles with larger macropores, enabling methylene blue to penetrate the interior. As the particle size increased and macroporosity decreased, adsorption becomes increasingly dominated by surface kinetics. In contrast, PD particles —which lack macropores—showed the opposite trend: smaller particles were primarily governed by fast surface adsorption, while in larger particles, diffusion through nanopores became increasingly relevant. Correlation analysis between adsorption rate constants and structural parameters such as particle diameter and pore sizes revealed strong, opposing trends. In PD particles, a near-perfect inverse correlation was observed between the diffusion and kinetic components, indicating competitive suppression, where the dominance of one mechanism limited the contribution of the other. These results demonstrated that internal pore architecture played a central role in controlling the adsorption mechanism. Tuning particle size and porosity allowed deliberate control over the balance between diffusion and surface kinetics, enabling the rational design of microparticle adsorbents with tailored uptake behavior for water purification and dye removal applications.

## 1. Introduction

The discharge of synthetic organic molecule pollutants, such as dyes—particularly cationic and anionic species—into aquatic environments poses serious ecological and health risks, as even low concentrations can lead to bioaccumulation, reduced photosynthetic activity in aquatic plants, and toxicity to fish, algae, and microorganisms [1,2]. Removal of the organic pollutants from the affected waters and ecosystems, efficiently and economically, is a current technological goal to sustainable environment [3,4]. The challenge is to design efficient adsorbents for organic pollutants, which require not only suitable surface chemistry but also a rational control over internal morphology, porosity, or size, affecting the mass transport properties to and from the adsorption sites. Among a wide range of materials sorbents, polymeric microparticles offer several advantages, making them attractive for the deployment for capturing and removal of the polluting small organic molecules from waters; these advantages include a high surface area, tunable chemical functionality, high stability, and scalable synthesis [3,4,5,6,7]. However, achieving precise control over their morphology and internal micro- and nanopore structures is critical to the transport of the reactants to the adsorption sites, and this remains a significant challenge. Traditional emulsion or precipitation polymerization methods often lead to poorly defined or inaccessible porosity, which has to be mitigated by second-stage polymerization and swelling [8,9].

Pickering emulsion polymerization, which uses solid particles as stabilizers, can lead to a successful path towards synthesis of porous adsorbents [10,11]. Porous polymer blocks, known as PolyHIPEs, can be obtained by polymerizing water-in-oil (w/o; where oil can be a monomer) high internal phase emulsions (HIPEs) [12,13]. The porosity of PolyHIPEs structures can be controlled with gradual addition of porogens or surfactants, which can break and thin the walls of the cells, enclose the water droplets, and lead to a large network of pores. Furthermore, polymerization of (monomer) oil-in-water (o/w) Pickering emulsions can lead to unique polymer adsorbents in the form of microparticles [11,14,15]. Recent advances in Pickering emulsion polymerization have demonstrated excellent control over hierarchical porosity in polymer microspheres [11,14,16], offering advantages in accessibility, monodispersity, and scalability compared to alternative methods, such as seeded swelling polymerization [8,9] or microfluidic emulsification. Pickering emulsion polymerization has recently proved to be extremely versatile [13,14,17] towards the production of polymer microsphere adsorbents, as it enables unique control over particle morphology, surface topology, and internal porosity by tailoring emulsion stability, monomer composition, and porogen content. However, relatively few experimental studies have demonstrated the great versatility of o/w Pickering emulsion polymerization technology to create polymer microparticles with tunable size and internal morphology—a critical aspect for developing efficient adsorbents.

In this study, we exploit o/w Pickering emulsion templating to synthesize two series of polymer microparticles with systematically varied internal morphology. We selected methacrylic acid (MA) as the functional monomer for its pH-responsive carboxylate groups that strongly favor electrostatic binding of cationic dyes like methylene blue (MB); ethylene glycol dimethacrylate (EGDMA) and divinylbenzene (DVB) as crosslinkers to modulate network flexibility, rigidity, and pore interconnectivity; and toluene as a porogen to gradually induce and tune pore formation. By systematically increasing the porogen (toluene) content and varying the crosslinking monomer chemistry, we produced particles exhibiting a gradual microporous to nanoporous architecture. Interestingly, the Pickering emulsion pore formation resembles the emulsion–solvent evaporation mechanism for pore creation in classical emulsions stabilized by surfactants, as reported by Li et al. [18]. Scanning electron microscopy (SEM) and pore size measurements confirmed a continuous shift in internal structure across the series. We then correlated these structural variations with the adsorption kinetics of MB. MB is widely used as a model cationic dye to study the sorption kinetics [19] of pollutants due to its high water solubility, strong UV-Vis absorbance, and well-characterized interaction with a variety of surfaces. Its adsorption behavior is highly sensitive to the structural features of the material, making it an ideal probe for elucidating the relationships between particle architecture and mass transfer mechanisms [20].

While previous studies have focused on the chemical modification of adsorbents [21,22], relatively few have addressed how architecture—specifically, the distribution and size of internal pores—governs the adsorption kinetic regime. Our work fills this gap by demonstrating that gradual morphological tuning leads to a switch in the dominant adsorption mechanism, and that the balance between diffusion and surface kinetics can be quantitatively linked to particle size and pore hierarchy. These insights provide a framework for the architecture-guided design of polymeric adsorbents, with relevance to broader applications in water treatment, separation, and catalysis. Adsorption of MB onto the microspheres is irreversible. Moreover, the adsorbed MB is protected from degradation by both hydrogen peroxide and UV light.

## 2. Results and Discussion

### 2.1. Preparation of Two Series of Oil-in-Water (O/W) Pickering Emulsions

Two series of Pickering emulsions were prepared by mechanically agitating an organic phase—composed of MA, a crosslinker (either EGDMA or DVB), and toluene—into an aqueous phase containing silica nanoparticles functionalized with 3-glycidyloxypropyltrimethoxysilane (NP-Gly) as emulsion stabilizers. To investigate the influence of the crosslinker type on the properties of the resulting emulsions, two distinct systems were designed: the PM series, containing EGDMA as crosslinker, and the PD series, containing DVB, as summarized in Table 1. Furthermore, to evaluate the role of toluene, which served as a solvent, reaction medium, and porogenic agent during polymerization, its volume fraction in organic phase was systematically varied from 0 to 1.25 mL in 0.25 mL steps increments for the PM series and from 0 to 1.0 mL in 0.25 mL steps for the PD series. For a 1.25 mL porogen the PD sample could not be obtained. Emulsions were formed by vigorous shaking of the organic and aqueous phases containing NP-Gly stabilizing nanoparticles. The emulsion compositions also contained 30 mg benzoin methyl ether (BME); 0.35 mL NP-Gly, 12 mL H_2_O; MA:EGDMA, and MA:DVB molar ratio was 10.6 all across the series.

**Figure 1:**
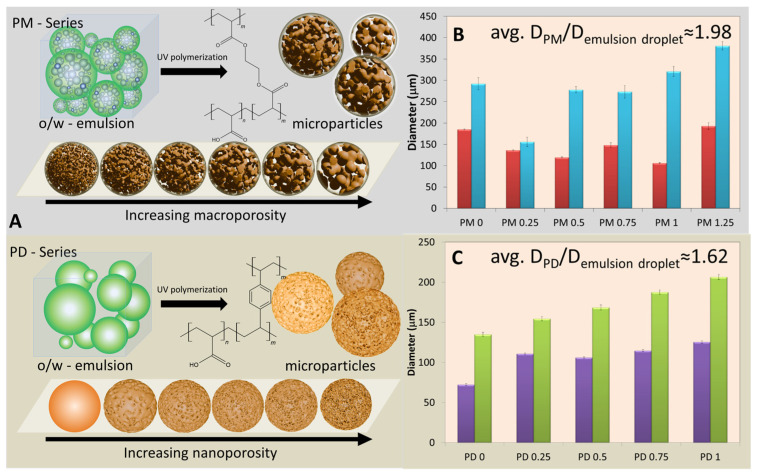
(**A**) Schematic representation of the UV-polymerization of o/w Pickering emulsions leading to the formation of polymer microspheres in the PM and PD series. (**B**) Histogram depicting the average droplet diameters in the precursor emulsions of PM series (red bars) and the corresponding polymerized microspheres of PM series (blue bars). (**C**) Histogram depicting the average droplet diameters in the precursor emulsions of PD series (purple bars) and the corresponding polymerized microspheres of PD series (green bars).

Optical and fluorescence microscopy images of the resulting emulsions from PM and PD series are shown in Figures S1 and S2, respectively, and the corresponding particle size distribution histograms are presented in Figures S3 and S4. The data revealed a distinct difference in how the two systems responded to the addition of toluene. In the PD series, the average droplet size of the organic phase increased steadily with increasing toluene content from 0.25 to 1 mL, consistent with the expected decrease in emulsion stability at higher solvent fractions. In contrast, the PM series exhibited a non-monotonic, oscillatory trend in droplet size. Specifically, the introduction of small to moderate amounts of toluene (≤1 mL) led to smaller droplet dimensions (e.g., compare PM 0 and PM 0.25 in Figure 1B), suggesting a stabilizing effect of toluene at low concentrations. However, at higher toluene volumes (>1 mL), this stabilization effect diminished, resulting in larger droplets. Such behavior can be interpreted in terms of variations in the immersion depth of the stabilizing nanoparticles at the oil/water interface. Changes in interfacial composition can significantly alter the nanoparticle contact angle at the three-phase boundary, thereby influencing emulsion stability, as amply discussed in different works [23,24]. Overall, these observations indicated that the addition of toluene exerted a stabilizing effect in the PM series emulsions, whereas in the PD series, toluene led to a monotonic increase in droplet diameter, as expected (Figure 1C).

### 2.2. Pickering Emulsion Polymerization—Synthesis of Microparticles

Following emulsion preparation, the Pickering emulsions were immediately subjected to photopolymerization. The polymer microspheres were synthesized according to previously reported general procedures [7,14]. The o/w Pickering emulsions were exposed to UV radiation and polymerized as illustrated schematically in Figure 1A. After polymerization, the Pickering emulsion droplets solidified into polymer microspheres exhibiting diverse sizes and morphologies, depending on the composition of the organic phase—specifically, the nature of the crosslinker and the toluene content.

The Pickering emulsions exhibited droplet diameters in the range of 105–192 μm for PM series emulsions (red bars in Figure 1B) and 72–125 μm for PD series emulsions (purple bars in Figure 1C), as determined by optical microscopy. Following polymerization, the resulting crosslinked microspheres showed comparable size ranges, with PM series particles of 155–380 μm (blue bars in Figure 1B) and PD series particles of 135–206 μm (green bars in Figure 1C), and with average diameters increasing systematically with the toluene porogen content. Particle and droplet diameters were measured from calibrated optical microscopy images using ImageJ software by analyzing >250 individual objects per sample for statistical reliability (detailed methodology in Section 3.3.2).

As shown in the schematics of Figure 1, increasing the amount of toluene in the organic phase in 0.25 mL increments produced a gradual enhancement in the level of internal porosity: predominantly microporous structures for the PM series (EGDMA-crosslinked) and nanoporous structures for the PD series (DVB-crosslinked). This increasing trend of porosity for both series is qualitatively illustrated in Figure 1A. The composition of the organic phase therefore played a decisive role in determining the internal morphology of the resulting polymer microparticles, while the choice of the crosslinker alone (EGDMA vs. DVB) dictated whether micropores or nanopores were predominantly formed, and this will be discussed in the following sections. Furthermore, this change in internal morphology was also reflected in the ratio between the average diameters of the polymerized microparticles and their precursor emulsion droplet diameters (Figure 1B,C). For instance, the average diameter ratio for the polymer microparticles in the PM series was 1.98—significantly higher than that of only 1.62 observed for the PD series—corroborating the more expanded, microporous morphology characteristic of the PM microparticles.

### 2.3. Microparticle Characterization

**Figure 2:**
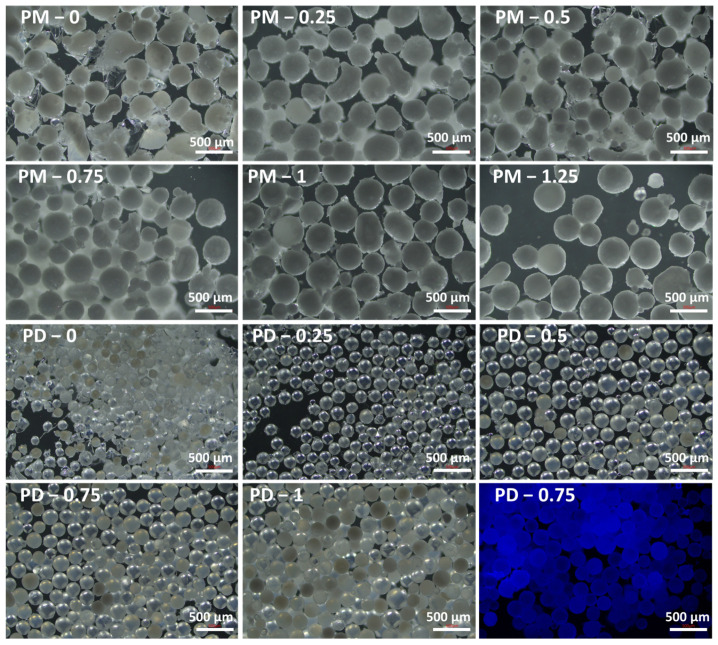
Optical and fluorescence microscope images of the obtained PM series and PD series of microparticles. The scale bar is 500 μm.

Optical microscopy images of the polymer microspheres from the PM and PD series synthesized via Pickering emulsion polymerization revealed a spherical morphology (Figure 2). Upon close inspection, the PM series microparticles appeared opaque, whereas those in the PD series were predominantly transparent and displayed very weak fluorescence under UV illumination. The opacity of the PM series particles was attributed to internal macroporosity, which enhanced light scattering, while the optical transparency of the PD series suggested a finer, nanoporous internal structure.

**Figure 3:**
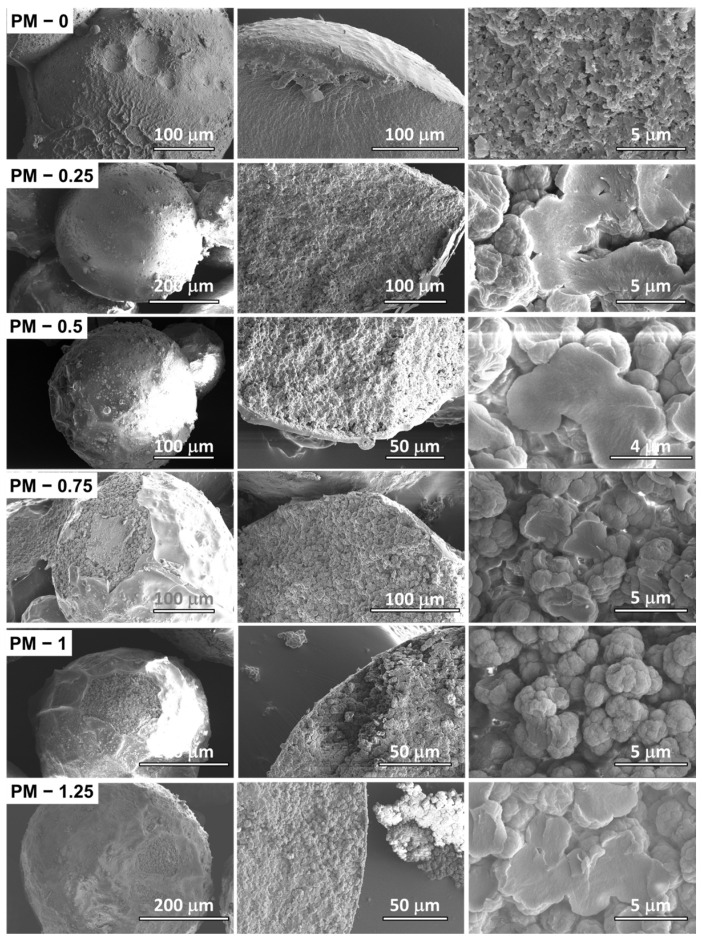
SEM images of the PM series microparticles synthesized with increasing toluene content in the organic phase. The left column shows the overall spherical morphology of PM microspheres (various magnifications from 200 to 350×), the middle column displays cross-section views (various magnifications from 500 to 800×), and the right column reveals the internal microstructure (various magnifications from 8000 to 12,000×).

The distinct internal morphologies of the two series of microparticles, as discussed in the previous section, were confirmed by SEM investigations. Representative SEM images of the PM series are presented in Figure 3. The PM microspheres exhibited well-defined spherical shapes with nanostructured outer surfaces, resulting from the self-assembly of NP-Gly nanoparticles at the oil–water interface used to stabilize the Pickering emulsion droplets. The organic phase of these Pickering emulsions droplets consisted of MA and EGDMA monomers with toluene as porogen, dispersed in an aqueous phase. Fractured microparticles revealed a highly granular internal structure, indicating the presence of interconnected micropores not stabilized by nanoparticles. An examination of the cross-section SEM images (800× magnification) in Figure 3 shows that all PM samples possessed this inner granular morphology, with the apparent grain size displaying an oscillatory evolution as the toluene content in the organic phase increased—initially increasing from PM 0 up to PM 0.25, then decreasing towards PM 1, and slightly increasing again for PM 1.25, consistent with the droplet size trends in Figure 1B. No clear correlation was observed between the average particle diameters of the PM series and the amount of toluene used in the organic phase of the Pickering emulsion. These observations suggested that the addition of toluene primarily influenced the internal microstructure rather than the external microparticle dimensions. The formation of a granular, microporous internal structure likely followed a mechanism similar to the emulsion–solvent evaporation proposed by Li et al. [18], where toluene migration into the aqueous phase and subsequent water diffusion inside the droplet during the microparticle formation and polymerization promoted internal micropore formation. However, the polymerization reaction appeared partially uncontrolled, as the pore and grain sizes did not exhibit a uniform dependence on toluene concentration.

**Figure 4:**
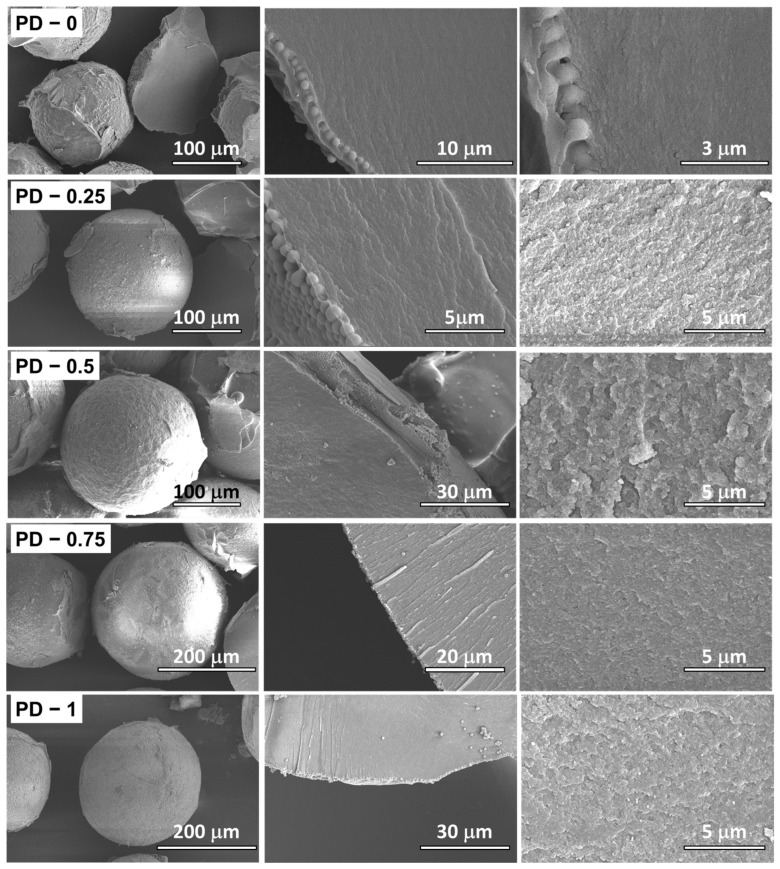
SEM images of the PD series microparticles synthesized with increasing toluene content in the organic phase. The left column shows the overall spherical morphology of the PD microspheres (various magnifications from 250 to 350×), the middle column displays cross-section views (various magnifications from 1500 to 8000×), and the right column reveals the internal nanostructure (various magnifications from 8000 to 15,000×).

In contrast to the PM series, the PD microparticles displayed a markedly different structural evolution. The average microparticle diameter increased monotonically with the toluene content in the organic phase, as shown in the histograms of Figure 1C. SEM images in Figure 4 reveal that the PD microparticles also possessed nanostructured outer surfaces due to self-assembly of NP-Gly nanoparticles at the oil–water interface during emulsification. Cross-section SEM micrographs at 800× magnification showed no evidence of internal macro grains, indicating the absence of microporosity; however, higher magnification (8000×) images clearly revealed the presence of nanopores within the polymer matrix. The Feret’s diameters of these nanopores increased systematically with the toluene content, as indicated in Figure S4, confirming the role of toluene as an effective porogen in the PD series of microparticles. For comparison, SEM analysis of the cross-section of the PM series of microparticles at similar magnifications (8000× and 12,000×) showed nanopores only in the first three compositions (Figure S5), after which the pores progressively closed and vanished completely at higher toluene contents. Considering the resolution limit of the FEG-SEM (≈1–2 nm at 12,000×), this observation suggested that residual pores in the later PM samples fell below the mesopore size regime (˂2 nm) and thus approached the lower detection limit of the technique.

### 2.4. Adsorption of Methylene Blue by Polymer Microparticles

The adsorption of MB onto the polymer microparticles is primarily governed by electrostatic interactions between the dye and the functional groups of the polymer surface. MB carries a positive charge on its dimethylamino groups, while the poly(methacrylic acid) (PMAA)-based microparticles expose negatively charged carboxylate groups. This electrostatic (Coulombic) attraction drives the rapid initial adsorption of MB onto the particle surface. The FTIR spectra of the microparticles before and after the adsorption confirm the encapsulation of MB; as seen in the discussion in the SI and the FTIR spectra in Figure S6. In addition to electrostatics, secondary interactions such as hydrogen bonding between the aromatic amine groups of MB and the carbonyl or hydroxyl moieties of the polymer, as well as π-π stacking between the aromatic rings of MB and the phenyl groups of DVB-crosslinked networks, may contribute to the overall adsorption process. The combined effect of these interactions can result in strong physical adsorption of MB on the polymeric matrices.

### 2.5. Adsorption Kinetics for Methylene Blue

Methylene blue serves as a widely used model cationic dye in adsorption studies. Its well-defined structure, strong UV-Vis absorbance, and sensitivity to surface chemistry make it an excellent probe for assessing adsorbent porosity, accessibility, and internal morphology. MB adsorption kinetics can also provide mechanistic insights relevant to removing structurally similar organic pollutants, aiding the current efforts in designing optimized polymeric adsorbents.

**Figure 5:**
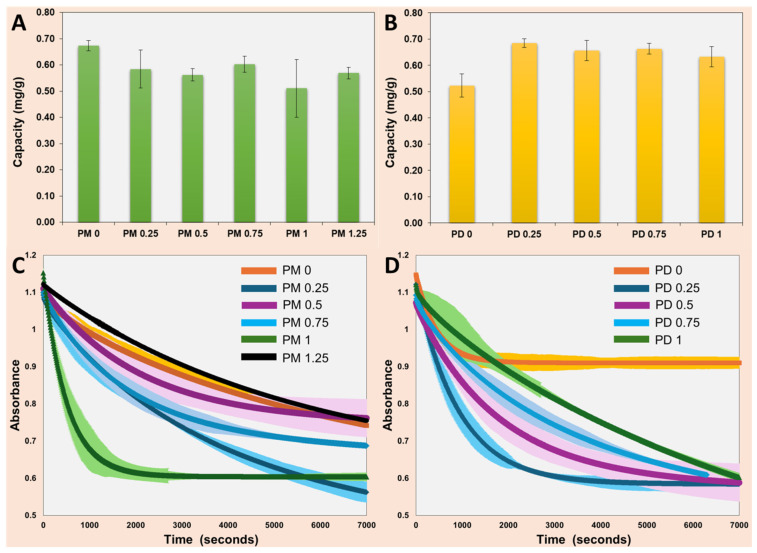
The adsorption capacity of the microparticles from the (**A**) PM series and (**B**) PD series. The absorbance vs. time of a MB stock solution (3.4 × 10^−5^ M) in water in the presence of particles from the (**C**) PM series and (**D**) PD series. The solid lines represent fitted curves through the experimental data points, averaged over at least three different measurements. The error bars represent the standard error from at least three measurements and are presented as a confidence interval around the main curves.

First, the adsorption capacity of both the PM and PD series of microparticles was evaluated using a concentrated MB stock solution (3.4 × 10^−5^ M) after 2 h of contact. In the PM series, a slight decrease in adsorption capacity was observed with increasing toluene content in the organic phase (Figure 5A), whereas the PD series of microparticles exhibited a modest increase in the adsorption capacity (Figure 5B). Overall, both systems displayed comparable adsorption trends, from 0.5 to 0.7 mg/g, with only minor variations in adsorption behavior within and between the series. The reported equilibrium adsorption capacities (q_e_) in this study were not the maximum adsorption capacities (q_max_) commonly reported in the literature following Langmuir isotherm fitting. The present work focused on elucidating structure–activity relationships through kinetic and morphological analysis rather than determining full saturation capacities via equilibrium isotherm studies. However, typical values reported for q_max_, for example, were for polydopamine microspheres (90.7 mg/g) [25], amphiphilic block copolymer microspheres (119.8 mg/g) [26], and cellulose nanocrystal–polymer composites (233 mg/g) [27], where chemistry (e.g., anionic sites) often dominated over morphology for cationic dyes like MB.

Next, both PM and PD series of microparticles were employed to investigate the adsorption kinetics of MB from the same stock solution (3.4 × 10^−5^ M). The decrease in the solution absorbance was monitored over a period of 2 h while PM or PD adsorbent microspheres were immersed in the MB solution. The microparticle adsorbents from each series were confined within a small stainless-steel mesh basket placed inside a standard 1 cm pathlength UV-Vis cuvette. Data acquisition was performed at 10 s time intervals. Continuous stirring was ensured by a 2 mm capsule shaped magnetic stir bar positioned directly inside the cuvette to maintain homogeneous mixing. Figure 5C,D show MB adsorption kinetics for the PM series and PD series microparticles, respectively. The profiles revealed that both adsorption rate and total uptake depended strongly on particle type, consistent with differences in internal morphology and porosity.

To gain deeper insight into the adsorption mechanism of MB onto the PM and PD microparticles, the experimental adsorption data for the MB dye were modeled using an equation that combines diffusion-controlled and first-order kinetic processes:

(1) $$C\left(t\right)=-x_{diff}\left(k_{diff}\sqrt{t}+C_{const}\right)+x_{kin}[-\left(\frac{A_{const}}{k_{kin}}\right)\left(1-\exp\left(-k_{kin}t\right)\right)+B_{const}],$$

where *C*(*t*) represents the observed dye concentration in solution (e.g., mol × L^−1^ or absorbance units, depending on detection method); *k_diff_* is the diffusion rate constant (s^−1/2^) associated with the square-root time dependence arising from Fickian transport into the porous structure; while *k_kin_* is the first-order kinetic rate constant (s^−1^) describing adsorption on internal surfaces; the dimensionless parameters *x_diff_* and *x_kin_* quantify the relative contributions of the diffusion-controlled and kinetic-controlled uptake processes, respectively, and satisfy *x_diff_* + *x_kin_* =1; the empirical constants *A_const_* and *B_const_* (same units as *C*(*t*)) define the amplitude and offset of the kinetic component; and *C_const_* is a constant background term (same units as *C*(*t*)) accounting for initial baseline concentration or detection offset. Equation (1) was selected as it effectively decoupled diffusion-controlled ($\sqrt{t}$ term) and surface kinetic contributions, the first-order exponential, in porous systems, commonly applied to dye adsorption on polymers [28].

The model assumed that, at short adsorption times, uptake was dominated by diffusion into the internal pore network, which followed the expected $\sqrt{t}$-scaling dependence characteristic of the cumulative adsorbed amount under semi-infinite Fickian diffusion. At longer times, the adsorption process transitioned to a surface-controlled regime governed by first-order adsorption kinetics. By fitting the contributions of each term via the weighting factors, *x_diff_* and *x_kin_*, the model enabled a mechanistic interpretation of the adsorption dynamics across different particle architectures. The fitted parameters obtained for both the PM and PD series, corresponding to the adsorption curves in Figure 5, are summarized in Table 2.

**Figure 6:**
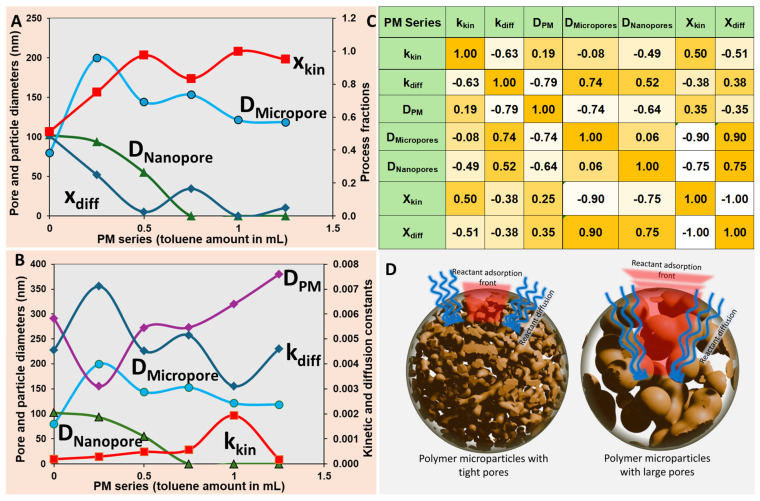
(**A**) Evolution of the diffusion (*x_diff_*) and kinetic (*x_kin_*) contribution fractions as a function of nanopore diameter (D_Nanopore_) and micropore diameter (D_Micropore_) (÷10) in the PM series. (**B**) Evolution of the diffusion (*k_diff_*) and kinetic (*k_kin_*) constants (on the secondary y-axis), the particle diameter (D_PM_) (÷1000), nanopore diameter (D_Nanopore_), and micropore diameter (D_Micropores_) (÷10) (on the primary y-axis) as a function of toluene amount in the PM series. (**C**) Color coded correlation table illustrating the relationships between the key parameters governing adsorption: strong positive (orange) or negative (white) correlations fall in the ±1 to ±0.7 range, moderate (yellow hues) correlations between ±0.7 and ±0.5, and negligible (yellow-white) correlations below ±0.5 values. (**D**) Schematic representation of the role of diffusion during MB adsorption in the macro- and nanoporous structure of PM microparticles.

Figure 6C illustrates the Pearson correlation coefficients (r) between the fitted kinetic parameters (*x_diff_*, *x_kin_*, *k_diff_*, *k_kin_*) and key structural parameters (nanopore diameter, macropore diameter, particle size). Strong opposing trends were evident, as *x_diff_* showed a positive correlation with nanopore diameter (r = +0.75), indicating that larger nanopores enhance the diffusion contribution, while *x_kin_* exhibited a strong negative correlation (r = −0.75), reflecting the suppression of surface kinetics as internal pore access improved.

In the case of the PM series of microparticles, the adsorption process evolved as a function of particle morphology. For PM 0, where no macropores were observed at 800× magnification, the fractional contributions of diffusion and kinetics were comparable (i.e., *x_diff_* ≈ *x_kin_*; Figure 6A). As macropores began to develop in the subsequent PM microparticles, the adsorption process shifted progressively toward a kinetics-dominated regime. The formation of larger internal domains increased the availability of subsurface adsorption sites, leading to enhanced MB fixation within the particle interior and a higher relative contribution of the first-order kinetic component.

The correlation maps presented in Figure 6C provide additional evidence clarifying the adsorption mechanism in the PM series:

- A strong positive correlation between *x_diff_* and nanopore size (r = +0.75) (*x_diff_*~D_nanopore_) and a strong negative correlation between *x_kin_* and nanopore size (r = −0.75) (*x_kin_*~1/D_nanopore_) indicated that diffusion was favored when nanopores were present and open, whereas the kinetic contribution increased as these nanopores progressively collapsed with higher toluene content (Figure 6A);
- Excluding PM 0, both *x_kin_* and the D_micropores_ showed a strong inverse correlation (r = −0.90), while *x_diff_* was strongly positively correlated with D_micropore_ (r = +0.90). This confirmed that the diffusion-controlled regime dominated when micropores were well developed, whereas the process became increasingly kinetic-controlled as the micropores shrank in response to higher toluene amount (Figure 6A);
- The diffusion constant *k_diff_* exhibited a strong inverse correlation with particle size (r = −0.79) (*k_diff_*~1/D_PM_), consistent with slower long-range transport in larger, less permeable particles;
- *k_diff_* also correlated positively with both micropore sizes (r = +0.74, *k_diff_*~D_micropores_) and nanopore sizes (r = +0.52, *k_diff_*~D_nanopores_), as shown in Figure 6C, supporting the previous conclusion that the diffusive regime dominated only when a hierarchical micro/nanoporous morphology was present. As pores collapsed at higher toluene amount, diffusion became less relevant and the kinetic, surface-limited regime increasingly prevailed;
- A further inverse correlation between *k_diff_* and *k_kin_* (r = −0.63) showed that these processes were not independent parallel pathways; instead, they competed for dominance, with diffusion decreasing as kinetic adsorption became more significant (Figure 6B);
- Finally, the inverse correlation between micropore diameter and particle size (r = −0.74) suggested that the overall microparticle size was intrinsically linked to the internal morphology, with larger PM microparticles tending to contain more compact or collapsed internal domains (Figure 6B).

These trends collectively indicated that diffusion remained dominant—or at least highly relevant—especially in the PM series samples exhibiting an internal micro- and nanoporous architecture. As these micro- and nanopores narrowed or collapsed with increasing toluene content, the ability of MB dye molecules to penetrate the PM microparticles interior became severely restricted, as illustrated schematically in Figure 6D. Mechanistically, a high *x_kin_* value indicated that adsorption occurred predominantly near the microparticle surface, leading to a reduced effective diffusion depth—a phenomenon known as “uptake front formation”.

Conversely, when diffusion was fast and MB could access deeper regions of the particle, kinetic adsorption may become limited either by the availability of surface sites or by their progressive saturation. Interestingly, while PM microparticles progressively shifted toward surface-controlled adsorption due to pore collapse and restricted internal transport, as we will see next, PD microparticles showed an increasing diffusion contribution as nanopore accessibility and particle size increase, leading to a transition toward mixed diffusion–kinetic control.

**Figure 7:**
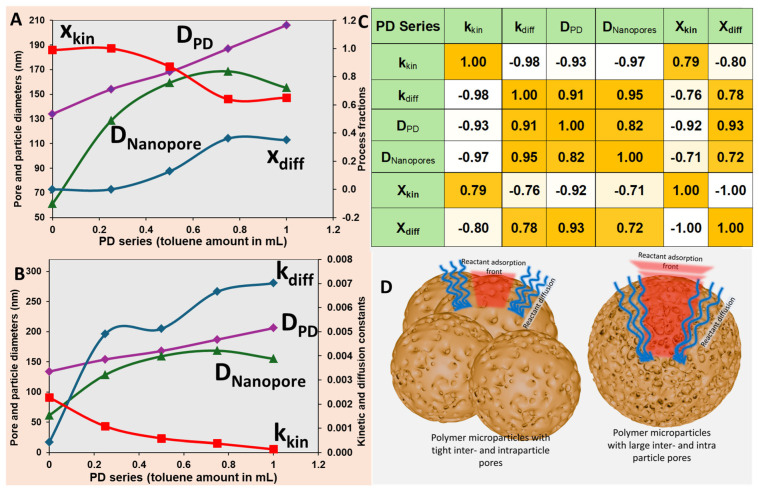
Adsorption mechanism of MB on PD microparticles series as a function of particle size and nanoporous architecture. (**A**) Evolution of the fractional contributions of diffusion-controlled (*x_diff_*) and kinetically controlled (*x_kin_*) adsorption with increasing toluene content in the PD series. (**B**) Evolution of the diffusion (*k_diff_*) and kinetic (*k_kin_*) rate constants (on the secondary y-axis) of PD particle diameter (D_PD_ ÷ 1000) and nanopore diameter (D_nanopore_) (on the primary y-axis) as a function of toluene amount in the PD series. (**C**) Color coded correlation matrix summarizing the relationship between adsorption parameters and morphological descriptors: correlation coefficients in the range ±1 to ±0.7 indicate strong direct (+, orange) or inverse (−, white) correlations, values between ±0.7 and ±0.5 indicate weak (yellow hues) correlations, and values between ±0.5 and 0 indicate very weak or negligible (yellow-white) correlations. (**D**) Schematic depicting the adsorption regimes in PD microparticles.

Figure 7C presents the Pearson correlations for the PD series, where nanopore diameter emerged as the dominant structural driver as *x_diff_* correlated strongly positively with nanopore size (r = +0.82), while *x_kin_* showed a marked negative correlation (r = −0.80). Particle diameter displayed a moderate positive link to diffusion dominance (r ≈ +0.55–0.65), consistent with larger PD particles, enabling more effective nanopore accessibility and reduced surface limitation. These opposing pore-size trends could highlight the competitive mechanisms which suggest nanoporosity is the key controlling factor.

In the case of the PD series of microparticles, which lacked micropores and were structurally dominated by a nanoporous internal architecture, the adsorption mechanism evolved distinctly with particle size. For the smaller PD particles (PD 0, PD 0.25), the process was kinetically controlled, as evidenced by *x_kin_* >> *x_diff_* and the high *x_kin_* value in Figure 7A. This behavior was expected because adsorption onto PD particles is a surface-dominated phenomenon, and smaller particles (PD 0, PD 0.25) offered a relatively higher external surface-area-to-volume ratio, enabling rapid binding of MB than the larger ones (PD 0.5, PD 0.75), according to the PD diameters presented in Figure 1C. The dominance of kinetics in this regime suggested that MB molecules were readily captured at or near the particle surface, likely via fast and irreversible binding, leading to uptake front formation. In such cases, internal diffusion (towards the interior of the particle) became largely irrelevant, as most dye molecules were adsorbed before they could penetrate deeper into the nanoporous network.

As the particle diameter increased with the increase in toluene amount in the PD series, the adsorption mechanism transitioned toward a more balanced regime, where *x_diff_* ≈ *x_kin_*, as seen in Figure 7A. This indicated that neither surface kinetics nor diffusion dominated exclusively, and both processes contributed significantly to the overall uptake rate. This shift may result from a decrease in accessible surface area per unit volume of the particles and the emergence of larger nanopores, which facilitated internal transport. Thus, the adsorption process relied more and more on the diffusion for the delivery and transport of the adsorbent molecule to the adsorption site.

The correlation map between adsorption process parameters and the morphological parameters of the PD series microparticles presented in Figure 7B bring additional insight.

- *x_diff_* showed a strong positive correlation with both particle diameter (r = +0.93) and nanopore size (r = +0.72), *x_diff_*~D_PD_~D_Nanopores_ (Figure 7C). This indicated that diffusion became increasingly significant as both the particle size and internal pore diameter increased;
- *x_kin_* exhibited strong negative correlation with particle diameter (r = −0.92) and nanopore size (r = −0.71), *x_kin_*~1/D_PD_~1/D_Nanopores_, indicating that kinetic contribution to the adsorption process became less relevant as interparticle and intraparticle diffusion pathways opened (Figure 7C);
- The diffusion rate constant *k_diff_* was strongly and positively correlated with both particle diameter (r = +0.91) and nanopore size (r = +0.95), *k_diff_*~D_PD_~D_Nanopores_, supporting the conclusion that diffusion efficiency increased as the nanopore architecture expanded inside larger PD microspheres, and as interparticle transport became less constrained (Figure 7D);
- Simultaneously, *k_kin_* showed a strong negative correlation with both particle size and nanopore diameter, *k_kin_*~1/D_PD_~1/D_Nanopores_, indicating that kinetic control weakened as internal diffusion pathways opened (Figure 7D).

In summary, smaller PD microparticles operated predominantly under kinetic control, governed by rapid surface adsorption, as reflected by the dominance of *x_kin_* >> *x_diff_* throughout the PD microparticles series (Figure 7A). As particle size and nanopore volume increased with higher porogen content, both inter- and intra-particle diffusion progressively contributed to the delivery of MB molecules to adsorption sites, leading to a mixed-control regime, especially for PD 0.75 and PD 1 microparticles. This highlighted a mechanistic transition from surface-limited adsorption to diffusion-assisted transport through the nanoporous network and interparticle spaces, consistent with the strong inverse correlation observed between *k_kin_* and *k_diff_* (r = −0.98).

### 2.6. Coupled Modeling of Diffusion and Surface-Driven Adsorption Kinetics

To quantitatively describe the transport and adsorption of dye molecules within the hierarchical porous polymer microspheres, a coupled model was developed that integrated both diffusion-controlled transport and adsorption kinetics. Each contribution was explicitly parameterized in relation to pore geometry and structural fractions extracted from experimental data, enabling a direct structure–function correlation. Starting from the phenomenological dual-process description used to fit the adsorption curves in Equation (1), the following analysis progressively reformulated the transport and adsorption terms into physically parameterized contributions linked to pore geometry and structural correlations.

#### 2.6.1. Diffusion Modeling Based on Hierarchical Porosity

To better understand which category of pores dominated the mass transport of the adsorbent toward internal adsorption sites, we modeled the total effective diffusion coefficient for each particle type as a weighted sum of three distinct contributions arising from different pore categories: diffusion through interparticle voids, micropores, and nanopores. The effective diffusion rate constant (s^−1/2^) can be expressed as:

(2) $$k_{diff,\;total}=w_{1}k_{diff,1}+w_{2}k_{diff,2}+w_{3}k_{diff,3}\;=\alpha_{d}\left(\frac{w_{1}\Delta_{1}}{D_{1}}+\frac{w_{2}\Delta_{2}}{D_{2}}+\frac{w_{3}\Delta_{3}}{D_{3}}\right),$$

where, for index *i*, 1 represents interparticle voids, 2 represents micropores, and 3 represents nanopores; $\alpha_{d}$ (m^−1^ × s^1/2^) is a scaling parameter; *D_i_* (m) are the corresponding interparticle voids, micropores, and nanopores diameters; the terms *k_diff,i_* (s^−1/2^) are the effective diffusion rate constants; Δ*_i_* (m^2^ × s^−1^) represents the effective diffusion coefficients associated with transport through interparticle voids, micropores, and nanopores, respectively; and *w_i_* corresponds to the volume fractions of each pore type within the total porosity (whereas the sum of all *w_i_* is normalized to 1). Further, the effective diffusion coefficients corresponding to each pore type are expressed as:

(3) $$\Delta_{i}=\frac{\Delta_{0}\times K\left(\lambda \right)}{\tau^{2}},$$

where τ is the tortuosity; Δ_0_ is the Fickian diffusion coefficient of MB through the solution, 7 × 10^−10^ m^2^ × s^−1^; and *K*(*λ*) is the diffusion hindrance factor for cylindrical pore, as calculated using the Renkin–Deen expression [29]:

(4) $$K\left(\lambda \right)={\left(1-\lambda \right)}^{2}\left(1-2.104\lambda +2.09\lambda^{3}-0.95\lambda^{5}\right)$$

where λ ≈ D_MB_/D_nanopore_, D_MB_ = 1.6 × 10^−9^ m (diameter of the MB).

Each diffusion pathway was constrained during model fitting to vary in a physically meaningful manner with respect to structural pore dimensions in the microparticle series. Specifically, *k_diff,_*_1_ was constrained to correlate with the interparticle void diameter, representing external transport channels throughout the PM series or PD series; *k_diff,_*_2_ was constrained to follow the trend of the micropore diameters, representing internal transport through mesoscopic channels throughout the PM series—for the PD series, this channel is absent; and *k_diff,_*_3_ was constrained to follow the trend of the nanopore diameters throughout the PM series or PD series, as seen in Figure 6C and Figure 7C, which dominated at the sub-micrometer scale and introduced confinement-limited diffusion.

#### 2.6.2. Kinetic Modeling Based on Internal Surface Area Scaling

We further extended the model to account for the adsorption kinetics under the assumption that the overall kinetic rate constant scaled proportionally with the total internal surface area accessible to dye molecules. This led to the following formulation for the kinetic rate constant (s^−1^):

(5) $$k_{kin,\;model}=w_{1}k_{kin,1}+w_{2}k_{kin,2}+w_{3}k_{kin,3}=\pi \alpha_{k}\left(w_{1}\frac{D^{3}}{D_{1}}+w_{2}\frac{D^{3}}{D_{2}}+w_{3}\frac{D^{3}}{D_{3}}\right),$$

where *k_kin,i_* are the effective kinetic rate constants (s^−1^) and $\alpha_{k}$ (m^−2^ × s^−1^) is a proportionality constant relating surface area to adsorption rate. The expression for each pore type arises from geometric considerations, assuming spherical symmetry and uniform distribution, the number of pores scaled as ∼$\frac{D^{3}}{D_{i}^{3}}$, and the surface area per void, micropore, or nanopore dimensions ∼$D_{i}^{2}$, resulting in a total area contribution proportional to $\frac{D^{3}}{D_{i}}$. The contribution of each pore category was thus weighted by its volume fraction $w_{i}$, derived from the diffusion model.

This kinetic formulation captured the physical reality that smaller pores, while occupying a limited volume, contributed disproportionately to the accessible surface area. Together, these models provided a structure–property framework that linked the multi-scale morphology of the particles to their functional performance in the capture of MB dye.

**Figure 8:**
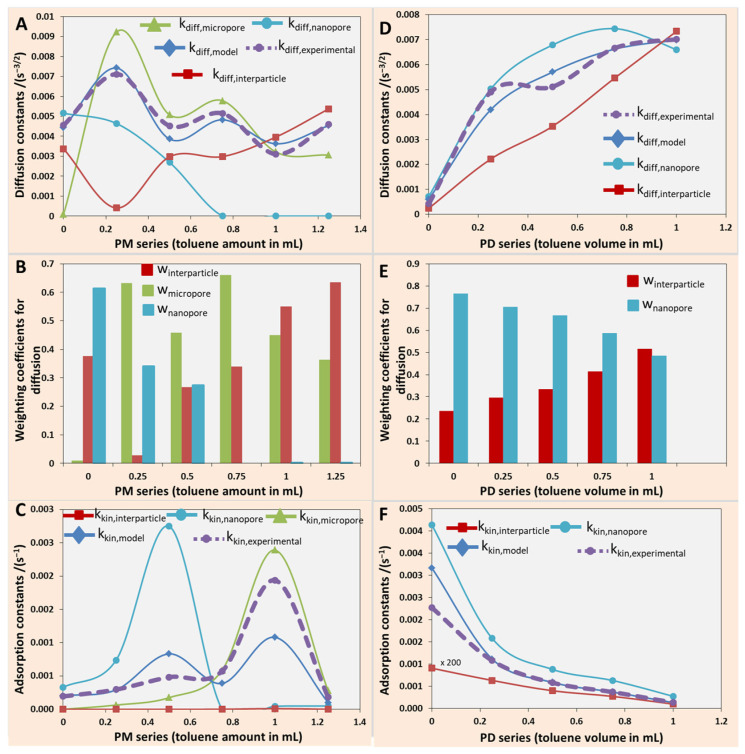
PM series: (**A**) Experimental k_diff,experimental_ and modeled k_diff,model_ diffusion constants decomposed into contributions from interparticle k_diff,interparticle_, micropore k_diff,micropores_, and nanopore k_diff,nanopores_ domains using Equation (2). (**B**) Weighting coefficients interparticle voids w_interparticle_, micropores w_micropores_, and nanopores w_nanopores_ for each pore class, reflecting their relative contribution to diffusion. (**C**) Experimental k_kin,experimental_ and modeled kinetic rate constants k_kin,model_, computed using Equation (5) based on internal surface area scaling of k_kin,nanopore_, k_kin,nanopore_, and k_kin,interparticle_. PD series: (**D**) Experimental and modeled diffusion constants using Equation (2). (**E**) Weighting coefficients for each pore class. (**F**) Experimental and modeled kinetic rate constants, computed using Equation (5), where the curve showing k_kin,interparticle_ has been multiplied by 200×.

*For PM series of microparticles:* as can be observed from Figure 8A, the experimental diffusion constants were accurately described by the model using Equation (2), which integrated the contribution of all pore types through fitted weighting coefficients and geometry-dependent diffusion parameters. The effective diffusion constant k_diff,model_ followed the experimental k_diff,experimental_ trend closely, particularly capturing the initial rise and the subsequent decay across the PM series. According to Equation (2), for the PM series, the modeled diffusion constant k_diff,model_ consisted of a weighted sum of three components, namely, interparticle diffusion, k_diff,interparticle_; diffusion through micropores, k_diff,micropores_; and diffusion through nanopores, k_diff,nanopores_. Notably, k_diff,nanopores_ dominated in the early stages of the series and became vanishingly small after PM 0.5 when the nanoporous structure closed. The k_diff,interparticle_ increased slightly in the PM series. However, most importantly, the diffusion through the micropores k_diff,micropores_ was dominant from PM 0.25 throughout the series, although the trend was oscillatory; it closely reflected the changes in micropore accessibility and connectivity as a function of synthesis conditions and the toluene amount, as shown in Figure 6.

Figure 8B shows the fitted weighting coefficients used in Equation (2), representing the relative volumetric contributions of interparticle voids w_interparticle_, micropores w_micropores_, and nanopores w_nanopores_ to the overall transport. A clear transition can be observed from a nanopore-dominated regime at low toluene content to an interparticle-dominated regime at higher toluene additions. This trend aligned with the expected structural evolution of the particles, in which increased toluene induced pore coarsening and network opening in the micropore network, as seen in Figure 6 and Figure S3. Further, micropore contributions remained significant throughout the series, peaking in the intermediate range where internal structuring appeared optimal for dye transport.

Figure 8C shows that the kinetic adsorption rate constants k_kin,model_ followed the experimental data k_kin,experimental_, indicating that the surface-area-weighted model described in Equation (5) had successfully captured the complexity. As shown in Figure 8C, the modeled curve matched the experimental profile, including the sharp increase in adsorption rate observed near 0.5 mL and 1 mL toluene. The first rise corresponded to the region where the nanopore surface area was maximized—k_kin,nanopore_ was maximum—as a consequence of the nanopores tightening, which then vanished as the nanopores closed and disappeared, as indicated by the weighting coefficients in Figure 8B. When the nanopores vanished from the structure > PM 0.5, the micropore surface area became dominant in the adsorption process, thus k_kin,micropore_ reached a maximum for the PM 1. In contrast, the interparticle contribution to the kinetic rate remained low and nearly invariant across the series, consistent with its limited surface area. The model confirmed that adsorption kinetics were strongly governed by the available internal surface area of different pore categories.

*For PD series of microparticles:* the experimental diffusion constants for the PD series were well captured by the model based on Equation (2) and are presented in Figure 8D. As shown, the modeled effective diffusion constant increased monotonically with the toluene volume, tracking the experimental data across the series. The nanopore diffusion term dominated the overall behavior, particularly at low to intermediate toluene volumes, while the interparticle diffusion contribution became increasingly relevant toward the end of the series. This trend reflected the progressive coarsening of the pore structure, in which interparticle voids expanded while also the nanopores increased in size. The fitted weighting coefficients extracted from the diffusion model in Equation (2), as seen in Figure 8E, indicated that nanopores were the dominant transport pathway throughout the early PD series, with volume fractions peaking around 0.25–0.5 mL toluene. The adsorption rate constants derived from experimental data were successfully modeled using Equation (5) and presented in Figure 8F. The kinetic rate decreased sharply with increasing toluene content, a trend attributed to the fact that the area became less available for the adsorbate as the area of interstitial void decreased with the particle size, and simultaneously, the area of the nanopore also decreased due to nanopore size increase. It is important to note that for both series, PM and PD, the contribution of the k_kin,interparticle_ component was rather negligible, clearly indicating that the adsorption of MB was mostly taking place inside of the pores of the microparticles, while microparticle surface area brought only a minor contribution to the overall process.

**Figure 9:**
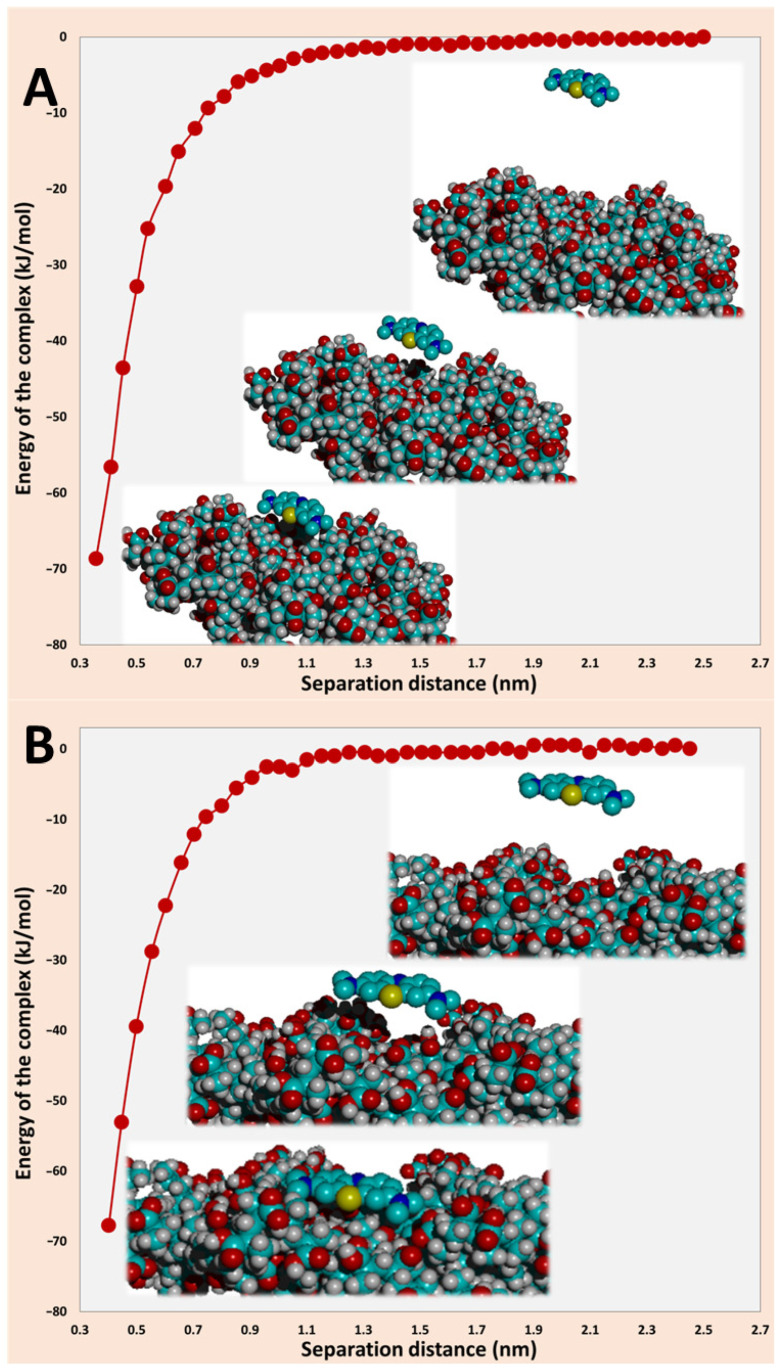
Calculated energy profiles for MB-polymer complex with separation distance from the surface of (**A**) PM microparticles and (**B**) PD microparticles. The color code for atoms is: cyan (Carbon), red (Oxygen), blue (Nitrogen), grey (Hydrogen) and yellow (Sulphur).

The adsorption of MB was modeled on PMAA slabs cross-linked with either EGDMA (PM series) or DVB (PD series), using the OPLS molecular mechanics force field to compute interaction energies. The detailed procedure was described in the Section 3. Briefly, both the adsorbate and the polymer slab were geometry optimized, after which the slab was frozen, while MB was allowed to relax. The optimized configuration revealed that MB adopted a parallel, slightly tilted orientation relative to the surface (Figure 9). Subsequently, the slab was unfrozen, and single-point energies of the complex were evaluated as a function of the vertical separation distance (d) by translating MB rigidly, preserving its orientation. The resulting adsorption energy profiles, as shown in Figure 9, exhibited a single potential minimum whose depth depended on the local adsorption site, with values of approximately −68 kJ mol^−1^ for MB on the PM slab and −67 kJ mol^−1^ for the PD slab. The equilibrium distance, corresponding to the minimum of the potential well, was found near 0.9–1.0 nm, consistent with non-covalent contact distances observed for dye–polymer interfaces.

The negative potential energy values confirmed that the adsorption process was thermodynamically favorable under the modeled (gas-phase) conditions. However, these potential energies should be regarded as enthalpic interaction energies, comparable in magnitude to experimental heats of adsorption, rather than Gibbs free energies, as entropic and solvation effects were not included in the molecular mechanics framework. In aqueous systems, such effects typically reduced the absolute magnitude of adsorption free energies by 30–60%, leading to experimental ΔG values in the range of −10 to −25 kJ mol^−1^ for MB on polymeric or oxide surfaces.

Indeed, reported Gibbs free energies of MB adsorption on polymeric materials spanned −11.6 to −46.8 kJ mol^−1^, with most studies attributing values between −10 and −20 kJ mol^−1^ to physisorption. For example, Polat et al. [30] reported Gibbs free energies between −5.4 and −16.0 KJ mol^−1^ for MB on functionalized PMMA composites, together with an enthalpy of 58.3 kJ mol^−1^, comparable in magnitude to our computed minimum potential energy on the PD slab (Figure 9B). Sakly et al. [31] found adsorption free energies of −11.6 to −18.7 kJ mol^−1^ on polyelectrolyte biopolymeric multilayers, while Djellali et al. [32] reported −14 kJ mol^−1^ for polyaniline surfaces. Ding et al. [33] observed slightly higher free energies (−19.2 to −21.7 kJ mol^−1^) for magnetic bio-based adsorbents, consistent with enhanced electrostatic contributions. In contrast, the adsorption of MB on carbon nanotubes, governed mainly by van der Waals forces, exhibited much weaker enthalpy (~7 kJ mol^−1^) [34]. The notably higher free energy (−46.8 kJ mol^−1^) reported by Aberkane et al. [35] for PVC-based nanocomposites suggested the onset of chemisorption involving partial charge transfer or covalent interactions.

Overall, the computed adsorption energies for the PM and PD slabs agreed well with experimental trends observed for MB on polar polymeric surfaces, confirming that electrostatic attraction between the cationic MB and the polymer surface was strong not only due to the presence of oppositely charged carboxylate groups that dominated the interaction, but also because of hydrogen bonding.

## 3. Materials and Methods

### 3.1. Materials

Ethylene glycol dimethacrylate (EGDMA, 97.5%), stabilized with hydroquinone monomethyl ether; divinylbenzene (DVB, 80% technical grade), stabilized with the same inhibitor; and methacrylic acid (MA, 99%) containing 250 ppm of 4-methoxyphenol were purchased from Sigma-Aldrich (Merck KGaA, Darmstadt, Germany). Prior to use, all compounds containing inhibitors were purified using an aluminum oxide (Al_2_O_3_) column. Tetraethyl orthosilicate (TEOS, 99%) and (3-glycidoxypropyl)trimethoxysilane (GLYMO) were also obtained from Sigma-Aldrich (Merck KGaA, Darmstadt, Germany). Benzoin methyl ether (BME, 97%) was acquired from ABCR GmbH (Karlsruhe, Germany). Toluene and absolute ethanol (EtOH, 99.3%) were supplied by Chemical Company (Iași, Romania). Ammonium hydroxide solution (NH_4_OH, 28–30%, EMSURE^®^ ACS, Reag. Ph Eur.) was purchased from Sigma-Aldrich (Merck KGaA, Darmstadt, Germany). Methylene blue (MB), high purity, biological stain, was purchased from Thermo Scientific Chemicals (Heysham, UK).

### 3.2. Synthesis Methods

The adsorbing polymeric microspheres were synthesized following a previously reported method [11]. In summary, the preparation of polymeric microspheres via Pickering emulsion polymerization involved three main steps: (a) synthesis of silica nanoparticles (NP-Gly) functionalized with 3-glycidyloxypropyltrimethoxysilane; (b) formulation of a Pickering emulsion using a mixture of monomer, crosslinker, porogen, and NP-Gly; and (c) UV radiation polymerization of the emulsion to obtain polymeric microspheres.

Two series of polymeric microspheres were generated by UV polymerization of Pickering emulsions, with the compositions outlined in Table 1 for the PM series and PD series. All mixtures were emulsified using a Vortex mixer (LLG, Lab Logistics Group GmbH, Meckenheim, Germany) at 3000 RPM for 1 min to generate oil-in-water droplets stabilized by NP-Gly. The Pickering emulsions were polymerized using a UV lamp (wavelength = 365 nm, with four lamps, each with an intensity = 2.2 mW cm^−2^) for 90 min. The resulting microparticles were isolated by filtration, thoroughly washed with ethanol to remove unreacted components, and dried at room temperature before further use. The two series of particles were denoted with PM (for those with EGDMA crosslinker) and PD (for those with DVB crosslinker), and an index was assigned from 0 to 1.25 or 0 to 1, respectively, corresponding to the amount of toluene used.

### 3.3. Characterization Methods

#### 3.3.1. Measurement of MB Adsorption Capacity of Microspheres

The MB adsorption capacity ($q_{MB}$) of the polymer microspheres was evaluated by UV-Vis spectrophotometry. A known mass (*m_P_*) of dried microspheres (typically 50 mg) was placed inside a small stainless-steel mesh basket and immersed directly into a glass cuvette containing 3.2 mL of aqueous MB stock solution of known initial concentration (*c_i_* = 3.4 × 10^−5^ M). The cuvette was sealed to minimize evaporation and kept under gentle magnetic stirring to maintain uniform dye concentration. The absorbance of the MB solution was monitored at 10 s intervals for 2 h at the maximum absorption wavelength (λ_max_ = 663 nm) using a UV-Vis spectrophotometer (DLAB Scientific Co., Ltd., Beijing, China). This configuration allowed in situ monitoring of MB adsorption by the polymer microparticles without removing samples from the cuvette, eliminating particle loss and ensuring reproducible kinetic measurements. The measurements for each type of particle were made in triplicate.

Next, the MB adsorption capacity $q_{MB}$ of microparticles was calculated with the following equation:

(6) $$q_{MB}=\frac{\left(c_{i}-c_{e}\right)\;V}{m_{P}},$$

where *c_i_* is the initial concentration of MB in the stock solution (mg × L^−1^); *c_e_* is the concentration of MB left in solution after being in contact for 2 h with the microparticles (mg × L^−1^); *V* is the volume of the sample (L), typically 3.2 mL; and *m_p_* is the dry mass of the microparticles (g).

#### 3.3.2. Scanning Electron Microscopy (SEM)

The microspheres were investigated with a Verios G4 UC (Thermo Fischer Scientific Inc., Eindhoven, The Netherlands) scanning electron microscope (SEM). The samples were coated by Pt/Au sputtering (≈8 Å thickness), and SEM imaging was performed using an accelerating voltage of 4–5 kV using an Everhart–Thornley detector with a 50 pA aperture. SEM observations were performed at multiple magnifications to resolve hierarchical features at different length scales. A magnification of 800× was used to evaluate macroporous morphology in the PM series (macropores were investigated but found absent in the PD series), as shown in Figure S3, while higher magnifications of 8000× and 12,000× were employed to examine nanoporous structures in both PM and PD microparticles, as shown in Figures S4 and S5, respectively. For the visual inspection of the particle morphology, the magnifications used for images in Figure 3 were: 200–350× (first column), 500–800× (second column) and 8000–12,000× (third column). Likewise, for the visual inspection of the particle morphology, the magnifications used for images in Figure 4 were: 250–350× (first column), 1500–8000× (second column) and 8000–15,000× (third column).

The diameters of the microparticles were measured by the corresponding Feret diameter of at least 250 particles, from calibrated images using ImageJ^TM^ software, version 1.54p.

The pore size distribution within the PM and PD series was determined from cross-sectional SEM micrographs using ImageJ^TM^ software, version 1.54p. Each image was first size calibrated and processed via a Fast Fourier Transform (FFT), and then a band-pass filter was applied (Process → FFT → Bandpass Filter, “Filter Large Structures” set to 30). The pores were then segmented by applying a grayscale threshold (Image → Adjust → Threshold) so that darker regions corresponding to voids were clearly highlighted. The equivalent (Feret) pore diameters were extracted by Analyze Particles procedure. Pore sizes represented mean equivalent Feret diameters from ImageJ analysis of calibrated SEM cross-sections (typical relative uncertainty ~10–20% for nanopores at these magnifications, consistent with literature on SEM-based porosity in polymers) [36].

#### 3.3.3. Fourier Transform Infrared (FTIR) Spectroscopy

Fourier transform infrared (FTIR) spectra of the polymeric microparticles were acquired using a DIGILAB-FTS 2000 Spectrometer (Bruker, Karlsruhe, Germany) over the 4000 to 400 cm^−1^ range, as seen in Figure S6.

#### 3.3.4. Optical and Fluorescence Microscopy Investigations

The microspheres were examined using an IM-5FLD inverted fluorescence microscope (Optika Srl, Ponteranica, Italy) equipped with an 8W XLED transmitted light illumination source, 5W LED excitation sources at 470, 560, and 385 nm, with corresponding blue, green, and UV filter sets, and a 6.3 MP Optika C-P6 color digital camera (Optika Srl, Ponteranica, Italy). Image acquisition and processing were performed using the OPTIKA PRO VIEW version 4.11.20805 software (Optika Srl, Ponteranica, Italy). All samples were analyzed under transmitted illumination using a 4× objective.

### 3.4. Computer Modeling

Molecular modeling simulations were performed using HyperChem Professional version 8.0, employing the Optimized Potentials for Liquid Simulations (OPLS) molecular mechanics force field. OPLS is well-suited for simulating molecular interactions in organic and polymeric systems due to its balanced treatment of non-bonded dispersion, electrostatics, and conformational energetics.

The polymer slab model was constructed from poly(methacrylic acid) (PMAA) crosslinked with either EGDMA or DVB at a ratio of one crosslinker per ten monomer units, resulting in structures containing approximately 2376 and 2444 atoms, respectively. Geometry optimization of each slab was conducted using the default parameters of the OPLS molecular mechanics force field. The MB molecule was built and energy-minimized independently. For adsorption modeling, MB was initially positioned above the polymer surface in a random, non-preferential orientation at different locations, at an initial vertical distance of less than 0.5 nm. The slab atoms were kept frozen while optimizing the geometry of MB. In all cases, MB spontaneously reoriented during minimization to adopt a nearly parallel or slightly tilted configuration, approaching the surface at a distance of ~0.35–0.4 nm. This relaxed orientation was subsequently maintained while the MB molecule was rigidly translated perpendicular to the surface in discrete steps (typically 0.05–0.5 nm), and single-point energy calculations were performed at each separation distance (*d*) with all slab atoms unfrozen.

The adsorption energy ($E_{ads}$) was calculated at each distance using the following expression:

(7) $$E_{ads}\left(d\right)=E_{complex}\left(2.5\;nm\right)-E_{complex}\left(d\right)$$

where $E_{complex}\left(d\right)$ is the total energy of the MB, the slab system’s separation distance is *d*, and the value at 2.5 nm was used as the non-interacting reference. All calculations were performed in the gas phase (implicit vacuum), without explicit solvent or long-range dielectric screening, to capture the intrinsic physisorption energy of MB on the polymer surface.

## 4. Conclusions

In this study, we demonstrated how controlled morphological tuning in polymer microspheres synthesized via Pickering emulsion polymerization governed both dye adsorption kinetics and the adsorption capacities. The combination of experimental modeling and molecular simulation established a clear structure–function relationship linking pore architecture, diffusional pathways, and surface chemistry.

The adsorption kinetics of methylene blue on the PM (EGDMA-crosslinked) and PD (DVB-crosslinked) series of microparticles were successfully described using a dual-component model combining diffusion-controlled and first-order kinetic terms. The combined experimental results and modeling demonstrated that pore accessibility critically governed the dominant adsorption regime. When micro- and nanopores progressively narrowed or closed, as observed in the PM series, adsorption became increasingly controlled by surface-limited kinetics, while the contribution of diffusion was strongly suppressed. In contrast, the opening of nanopores in the PD series enabled access to internal adsorption sites within the microparticles, thereby enhancing diffusive transport and increasing the relative contribution of diffusion-controlled uptake. Overall, the PD series exhibited faster initial MB uptake and slightly higher adsorption capacity compared to the PM series, owing to its more accessible nanoporous structure that favored diffusion-assisted transport. In contrast, pore narrowing in the PM series progressively shifted adsorption toward surface-controlled kinetics, limiting internal diffusion and reducing uptake efficiency. Eventually, this was also reflected in the slightly more favored adsorption capacity for the PD series vs. PM series. Further studies should focus on investigating the engineering aspects of deploying these materials as adsorbents, either as standalone systems or in composites, to avoid secondary plastic pollution.

Molecular mechanics simulations using the OPLS force field provided complementary microscopic insight into adsorption energetics. The computed potential energy profiles exhibit minima of approximately −68 kJ·mol^−1^ for MB on PM and −67 kcal·J^−1^ for MB on PD slabs, corresponding to moderately strong physisorption dominated by electrostatic interactions between the cationic MB and the polar ester or carboxylate functionalities of the polymer. These values were consistent in magnitude with experimentally reported enthalpies of MB adsorption on polymeric matrices. Although these computational results indicated comparable adsorption strengths for MB on both PM and PD slabs, it is clear that the adsorption rates and adsorption capacities were mostly controlled by the adsorption site availability across the particle series, which was well reflected in the kinetics and diffusion rate constants, as well as in the adsorption capacities, favoring the PD series.

Overall, this combined approach has established that not only surface chemistry but also the particle morphology can control the MB dye uptake, highlighting the versatility of Pickering emulsion polymerization methods to achieve gradual control in polymer microparticle morphology.

## Figures and Tables

**Table 1:** The composition of the PM series and PD series of Pickering emulsions stabilized by NP-Gly nanoparticles.

PM Series	Composition	PM 1.25	PM 1	PM 0.75	PM 0.5	PM 0.25	PM 0
	EGDMA (mL)	0.99	0.99	0.99	0.99	0.99	0.99
	MA (mL)	2.5	2.5	2.5	2.5	2.5	2.5
	Toluene (mL)	1.25	1	0.75	0.5	0.25	0
PD Series	Composition	-	PD 1	PD 0.75	PD 0.5	PD 0.25	PD 0
	DVB (mL)	-	0.5	0.5	0.5	0.5	0.5
	MA (mL)	-	2.5	2.5	2.5	2.5	2.5
	Toluene (mL)	-	1	0.75	0.5	0.25	0

**Table 2:** Values of the fit parameters to the adsorption curves according to Equation (1), together with the diameter of the microparticles from the PM series (D_PM_) and PD series (D_PD_), and the diameters of the nanopores (D_nanopore_) and micropores (D_micropores_).

PM series							
	*k_diff_* (s^−3/2^)	*x_diff_*	*k_kin_* (s^−1^)	*x_kin_*	D_PM_ (μm)	D_nanopore_ (nm)	D_micropore_ (nm)
PM 0	0.0046	0.4867	0.0002	0.5120	291	102	960
PM 0.25	0.0071	0.2500	0.0003	0.7500	155	93	1953
PM 0.5	0.0045	0.0256	0.0005	0.9744	272	55	1937
PM 0.75	0.0051	0.1660	0.0006	0.8330	273	-	1890
PM 1	0.0031	0.0000	0.0019	0.9982	320	-	1219
PM 1.25	0.0046	0.0500	0.0002	0.9500	380	-	1559
PD series							
	*k_diff_* (s^−3/2^)	*x_diff_*	*k_kin_* (s^−1^)	*x_kin_*	D_PD_ (μm)	D_nanopore_ (nm)	D_micropore_ (nm)
PD 0	0.0004	0.0000	0.0023	0.9888	134	61	-
PD 0.25	0.0049	0.0000	0.0011	1.0000	154	128	-
PD 0.5	0.0051	0.1270	0.0006	0.8720	168	159	-
PD 0.75	0.0067	0.3605	0.0004	0.6396	187	169	-
PD 1	0.0070	0.3495	0.0001	0.6506	206	155	-

## Data Availability

The original contributions presented in this study are included in the article/Supplementary Materials. Further inquiries can be directed to the corresponding author.

## Supplementary Materials

The following supporting information can be downloaded at: https://www.mdpi.com/article/10.3390/ijms27062591/s1.
