# Supplementary material for: Gradual Morphological Tuning in Polymer Microspheres via Pickering Emulsion Synthesis: Architecture-Controlled Dye Adsorption and Encapsulation
Source: Int J Mol Sci. 2026 Mar 12;27(6):2591. doi: 10.3390/ijms27062591 (PMC13026523; doi:10.3390/ijms27062591)
Supplement: Supplementary file 1 [file ijms-27-02591-s001.zip › ijms-4162188-supplementary.pdf]

Supporting Information

Gradual Morphological Tuning in Polymer Microspheres via Pickering Emulsion Synthesis: Architecture-Controlled Dye Adsorption and Encapsulation

Mirela Honciuc*, Oana-Iuliana Negru, Andrei Honciuc

"Petru Poni" Institute of Macromolecular Chemistry, 41A Grigore Ghica Voda Alley, Iasi, 700487, Romania

* Correspondence: honciuc.mirela@icmpp.ro

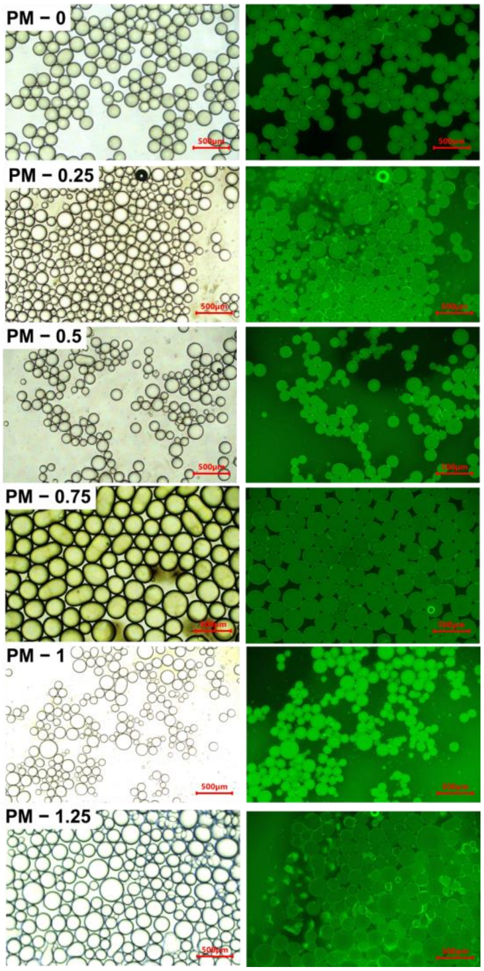

Figure S1. Optical and fluorescence microscope images of o/w Pickering emulsions for PM-series, stabilized by NP-Gly nanoparticles, showing marked difference in emulsion droplet sizes. The organic phase contains Hostasol Yellow 3G dye.

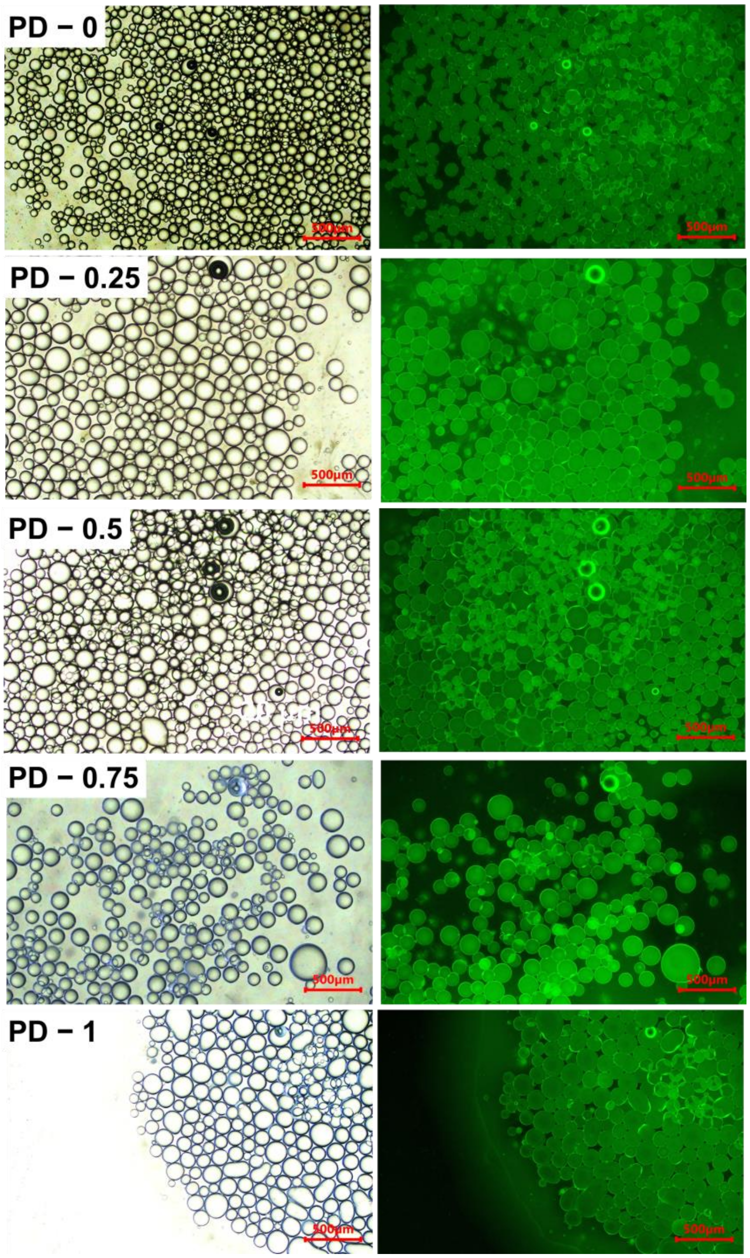

Figure S2. Optical and fluorescence microscope images of o/w Pickering emulsions for PD-series, stabilized by NP-Gly nanoparticles, showing marked difference in emulsion droplet sizes. The organic phase contains Hostasol Yellow 3G dye.

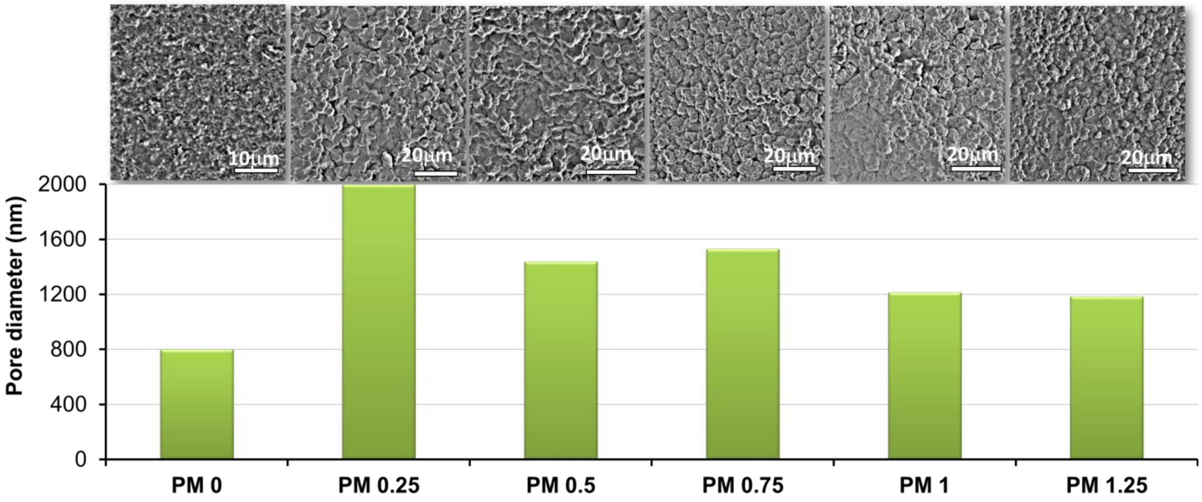

Figure S3. SEM images at 800× magnification and histogram showing the evolution of the Feret's diameter of the macropores in the PM-series of microparticles.

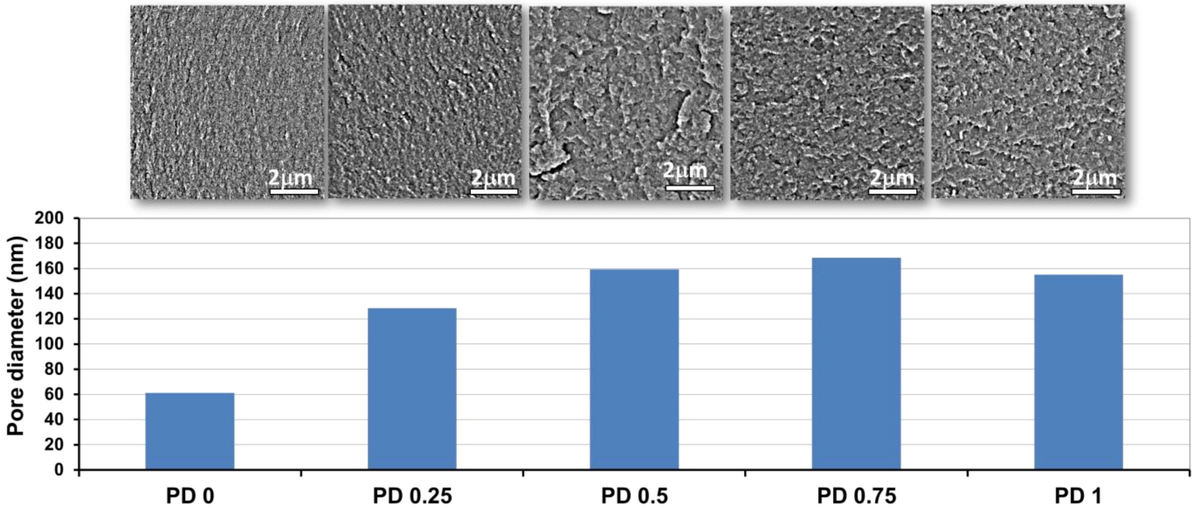

Figure S4. SEM images at 8000× magnification and histogram showing the evolution of the Feret's diameter of the nanopores in the PD-series of microparticles.

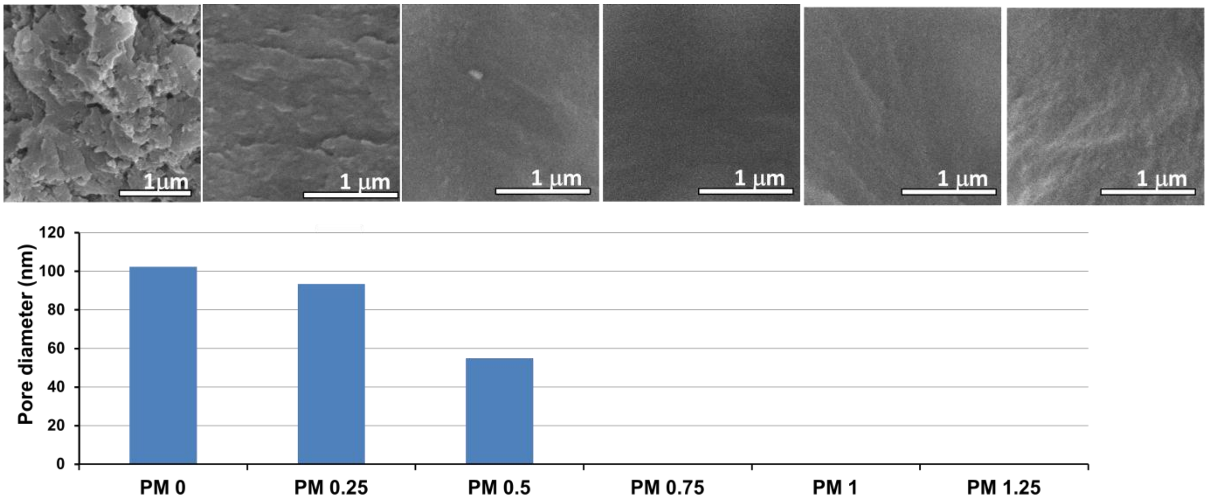

Figure S5. SEM images at 12000× magnification and histogram showing the evolution of the Feret's diameter of the nanopores in the PM-series of microparticles.

The FTIR spectra in **Figure S6** confirm successful adsorption of MB onto the PM 0.75 microspheres. The pure MB spectrum (orange) displays two relevant bands at 1604 cm^{-1} and 1570 cm^{-1}, attributed to C=N stretching in the central heterocyclic ring (coupled with aromatic C=C) and aromatic C=C/C-N skeleton vibrations of the phenothiazine core, respectively. These bands are absent in the bare PM 0.75 microspheres (green), which instead show a strong carbonyl C=O stretch at 1708 cm^{-1} from the methacrylic acid/ester groups. After MB adsorption (blue spectrum), the same 1604 cm^{-1} and 1570 cm^{-1} bands appear clearly, overlapping with the polymer matrix features but retaining their positions with minimal shift, indicating physisorption dominated by electrostatic interactions between cationic MB and anionic carboxylate sites on the PM particles, rather than covalent bonding. The emergence of these MB-specific vibrations provides direct spectroscopic evidence of dye uptake and supports the observed adsorption behavior in the kinetic and capacity studies.

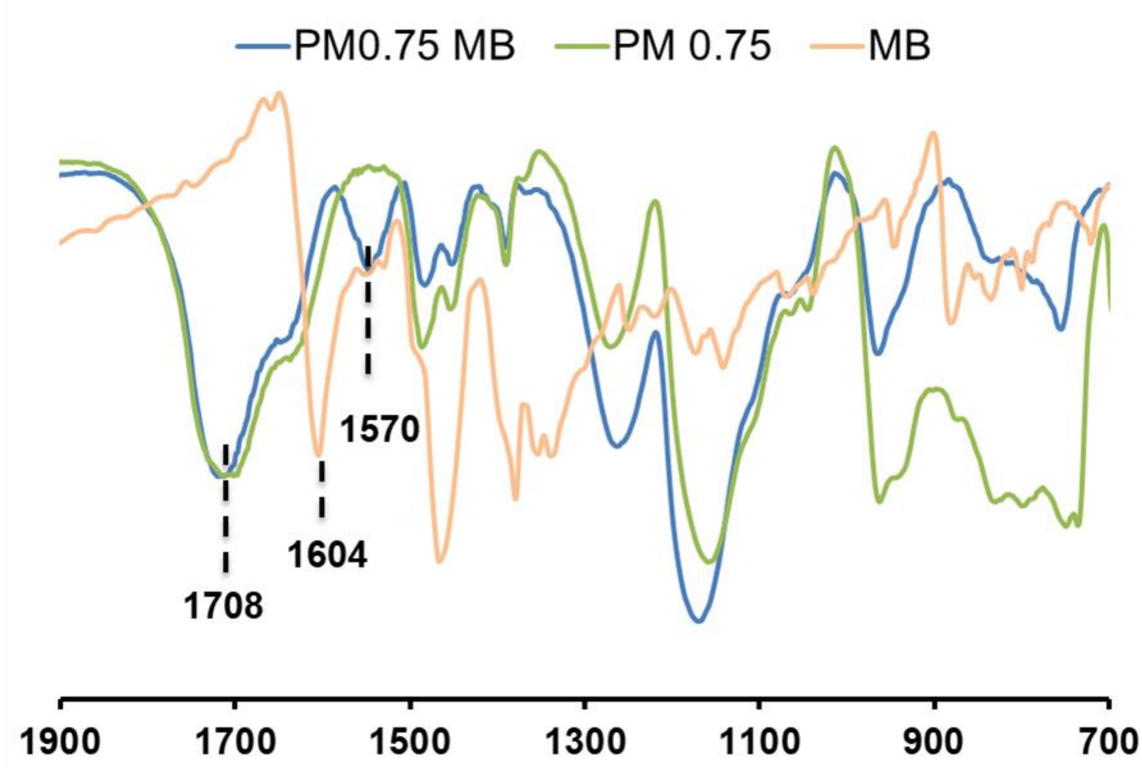

Figure S6. FTIR spectra of the MB, PM 0.75 microparticles and MB encapsulated microparticles.
